# Optimization of Poly-γ-Glutamic Acid Production by Moderate Electric Field-Assisted Fermentation in a *Haematococcus pluvialis*-Based Medium

**DOI:** 10.3390/microorganisms14081732

**Published:** 2026-08-07

**Authors:** Beom-Su Cho, Junho Yang, Min-Gyu Park, Jin Hong Mok, Jiyoung Shin, Ji-Young Yang

**Affiliations:** 1 Department of Food Science and Technology, Pukyong National University, 45 Yongso-ro Nam-gu, Busan 48513, Republic of Korea; jbs4644@gmail.com (B.-S.C.); kkandol83@naver.com (M.-G.P.); 2 Institute for Agricultural Product Quality and Safety Assessment, Kyungpook National University, 80 Daehak-ro, Buk-gu, Daegu 41566, Republic of Korea; jh.yang@knu.ac.kr; 3 Department of Food Science and Biotechnology, Dongguk University-Seoul, 32 Dongguk-ro, Ilsandong-gu, Goyang 10326, Republic of Korea; jhmok1024@dgu.edu

**Keywords:** *Haematococcus pluvialis*, optimization, *Bacillus subtilis*, poly-γ-glutamic acid, moderate electric field, fermentation

## Abstract

Moderate electric field (MEF) fermentation was investigated as a nonthermal stress strategy to enhance the formation of extracellular polymeric substances (EPS; mucilage) by *Bacillus subtilis* HA in a *Haematococcus*-based medium. Response surface methodology (RSM), combined with a Box–Behnken design, was employed to optimize the fermentation medium composition and the MEF operating parameters. Under conventional fermentation (CF), the optimized medium (2.91% *Haematococcus pluvialis*, 3.10% glucose, and 13.42% L-glutamate) yielded 8.60 ± 0.25% mucilage, closely matching the predicted value (8.54%) and confirming model adequacy. The optimized MEF conditions (85.2 Hz, 0.29 A, and 40.90% duty cycle) increased mucilage formation to 13.59 ± 0.18%, corresponding to approximately 1.6-fold enhancement compared with CF. Statistical analysis indicated that frequency–current interactions and a nonlinear duty cycle response governed EPS accumulation. Substrate–metabolite analysis further showed that MEF significantly reduced residual L-glutamate (1.25 ± 0.03%) while increasing poly-γ-glutamic acid (γ-PGA) content (11.07 ± 0.16%), indicating enhanced conversion of L-glutamate into γ-PGA-rich mucilage. In comparative physicochemical stress experiments, 3% KCl supplementation produced the highest mucilage formation among non-MEF conditions. The MEF-treated system achieved the highest mucilage production, indicating that MEF is a tunable and effective strategy for promoting substrate-to-polymer conversion and regulating EPS production in *Bacillus*-based fermentation systems.

## 1. Introduction

Microalgae have gained increasing attention as a sustainable biological resource with a rich content of proteins, amino acids, carbohydrates, and other essential nutrients [1]. Beyond their conventional use as functional foods or dietary supplements [2,3], their well-balanced biochemical composition renders them promising alternative substrates for microbial fermentation, offering a route to convert microalgal biomass into value-added metabolites [4].

Among edible microalgae, *Haematococcus pluvialis* is one of the richest natural sources of astaxanthin, containing approximately 3–5% on a dry weight basis [5]. Astaxanthin derived from *H. pluvialis* exhibits potent biological activities, including antioxidant, anti-inflammatory, antidiabetic, and anticancer effects [6], and is therefore widely used across the food, cosmetic, and pharmaceutical industries. In addition to its value as an astaxanthin-producing microalga, *H. pluvialis* may serve as a nutrient-rich medium component for producing value-added microbial metabolites. Therefore, the use of *H. pluvialis*-based media could provide a sustainable approach for linking microalgal biomass utilization with microbial biopolymer production.

*Bacillus* is a genus of spore-forming Gram-positive bacteria traditionally used in fermented soybean foods such as cheonggukjang and natto, and is widely applied for the production of enzymes and bioactive compounds [7]. Among its metabolites, poly-γ-glutamic acid (γ-PGA) is a unique biopolymer composed of D- and L-glutamic acid units linked by α-amino/γ-carboxyl amide bonds [8], and its edible, water-soluble, and biodegradable nature makes it a high-value biomaterial for food, cosmetic, and biomedical applications [9], with additional physiological benefits such as anticancer effects and enhanced intestinal calcium absorption [10]. Accordingly, extensive efforts have been made to maximize γ-PGA production through metabolic pathway regulation and fermentation optimization [11]. However, γ-PGA production remains strongly dependent on strain characteristics, medium composition, and environmental conditions, and the viscosity increase associated with extracellular polymer accumulation often limits process efficiency. Applying sublethal stressors—such as salt or pH modulation—has been shown to markedly enhance γ-PGA yield [12,13] by inducing metabolic and cellular reorganization of extracellular polymeric substances (EPS) [14]. Our recent study confirmed that *H. pluvialis* serves as a viable fermentation matrix for *B. subtilis* HA, supporting γ-PGA-rich mucilage accumulation under MSG supplementation [15]. In the present study, mucilage refers to the crude extracellular viscous fraction produced during B. subtilis HA fermentation, in which γ-PGA is the principal polymeric component together with minor amounts of proteins and carbohydrates.

To promote an efficient stress response, moderate electric field (MEF) treatment was introduced as an effective stress stimulus to enhance *B. subtilis* fermentation. MEF is a food processing technology that applies an electric field below 1 kV/cm. When an electric field is applied to biological cells, a transmembrane potential difference is generated, leading to increased cell permeability. Under high-temperature conditions, MEF operates according to the principles of ohmic heating, whereas under controlled low-temperature conditions, it functions as a nonthermal technology with minimal thermal effects. The application of MEF in food science has been primarily limited to microbial inactivation technologies [16], and studies investigating MEF as a synergistic enhancer of microbial fermentation remain scarce. Thus, MEF can be considered a physical process-intensification strategy that may influence microbial fermentation performance by affecting membrane permeability and cellular transport under controlled nonthermal conditions.

Although MEF has been applied in various food processing fields, its use as a fermentation-enhancing strategy for γ-PGA-rich mucilage production by *B. subtilis* in *H. pluvialis*-based medium remains largely unexplored. Whereas [15] modulated γ-PGA through nutritional means, the present study introduces MEF as a physical stressor targeting membrane-associated transport. The aim of this study was to evaluate the feasibility of MEF-assisted fermentation for enhancing γ-PGA-rich mucilage production by *B. subtilis* HA in *H. pluvialis*-based medium, optimize MEF-assisted fermentation parameters, and evaluate its potential as a fermentation enhancement strategy.

## 2. Materials and Methods

### 2.1. Materials and Chemicals

The *Haematococcus* extract powder used in this study was purchased from EOM-MA AESON Co., Ltd. (Gimcheon, Republic of Korea). Monosodium L-glutamate (MSG) and glucose, which were used as substrates for mucilage production, were purchased from CJ CheilJedang Co., Ltd. (Seoul, Republic of Korea) and Q-One Co., Ltd. (Seoul, Republic of Korea), respectively. Skim milk, used as a medium for starter culture preparation intended for food applications, was obtained from the Seoul Milk Cooperative (Seoul, Republic of Korea). MRS broth was purchased from Difco^TM^ Lactobacilli MRS (Becton, Dickinson and Company, Sparks, MD, USA). All other reagents used in the analyses were of analytical grade.

### 2.2. Strains and Preparation of Culture Starter

To activate the strain, *B. subtilis* HA (KCCM 10775P), a generally recognized as safe strain isolated from Cheonggukjang, was streaked onto MRS agar and cultured at 37 °C for 24 h. A single loopful of the culture was inoculated into sterilized 5% skim milk medium and incubated at 37 °C for 24 h with shaking at 160 rpm to prepare the starter culture.

### 2.3. Experimental Design and Optimization for Mucilage Production

In this study, response surface methodology (RSM) was used using Design Expert Version 23.0 (Stat-Ease Inc., Minneapolis, MN, USA) to construct the experimental design, predict the optimal fermentation medium and MEF-fermentation conditions for maximum mucilage production, and perform data analysis and model development. Two Box–Behnken designs (BBDs) were implemented in this study.

#### 2.3.1. Optimization of *Haematococcus*-Based Medium

To optimize the fermentation medium conditions, three independent variables (*H. pluvialis*, glucose, and L-glutamate) were selected, and mucilage concentration was used as the dependent variable. Each independent variable was coded at three levels (−1, 0, and +1), corresponding to the minimum, center, and maximum values, respectively. The corresponding values were established through single-factor experiments. The dependent variable was defined as the mucilage content determined using a mucilage assay (Table S1). To ensure the reproducibility and reliability of the model, 17 experimental runs were performed in a randomized order, including five replicates at the center point. Following experimentation, a second-order polynomial model was developed through statistical analysis, and this predictive model was validated by comparing its outputs with experimentally measured values under statistically optimized conditions.

#### 2.3.2. Optimization of MEF-Assisted Fermentation

Based on the optimal fermentation medium conditions derived in Section 2.3.1, RSM-BBD was used to optimize the MEF-fermentation conditions. Frequency, current intensity, and duty cycle were selected as independent variables, and mucilage concentration was used as the dependent variable. To control temperature effects, the non-ohmic heating region was determined through single-factor experiments.

Each independent variable was encoded at three levels (−1, 0, and +1), representing the minimum, center, and maximum settings, respectively. The frequency variable was defined on a logarithmic scale to enable a comparison between the low- and high-frequency regions (Table S2).

### 2.4. Fermentation Process

#### 2.4.1. Conventional Fermentation

The fermentation medium was prepared in a 300 mL baffled reaction vessel, and the concentrations of *H. pluvialis*, glucose, and MSG were adjusted according to the compositions listed in Table S1. *B. subtilis* HA starter was inoculated at a final concentration of 50 g·L^−1^, and the total volume was standardized to 150 mL with sterilized distilled water. The nutrient solution was incubated at 37 °C with shaking at 160 rpm for 48 h.

#### 2.4.2. MEF-Assisted Fermentation

**Figure 1:**
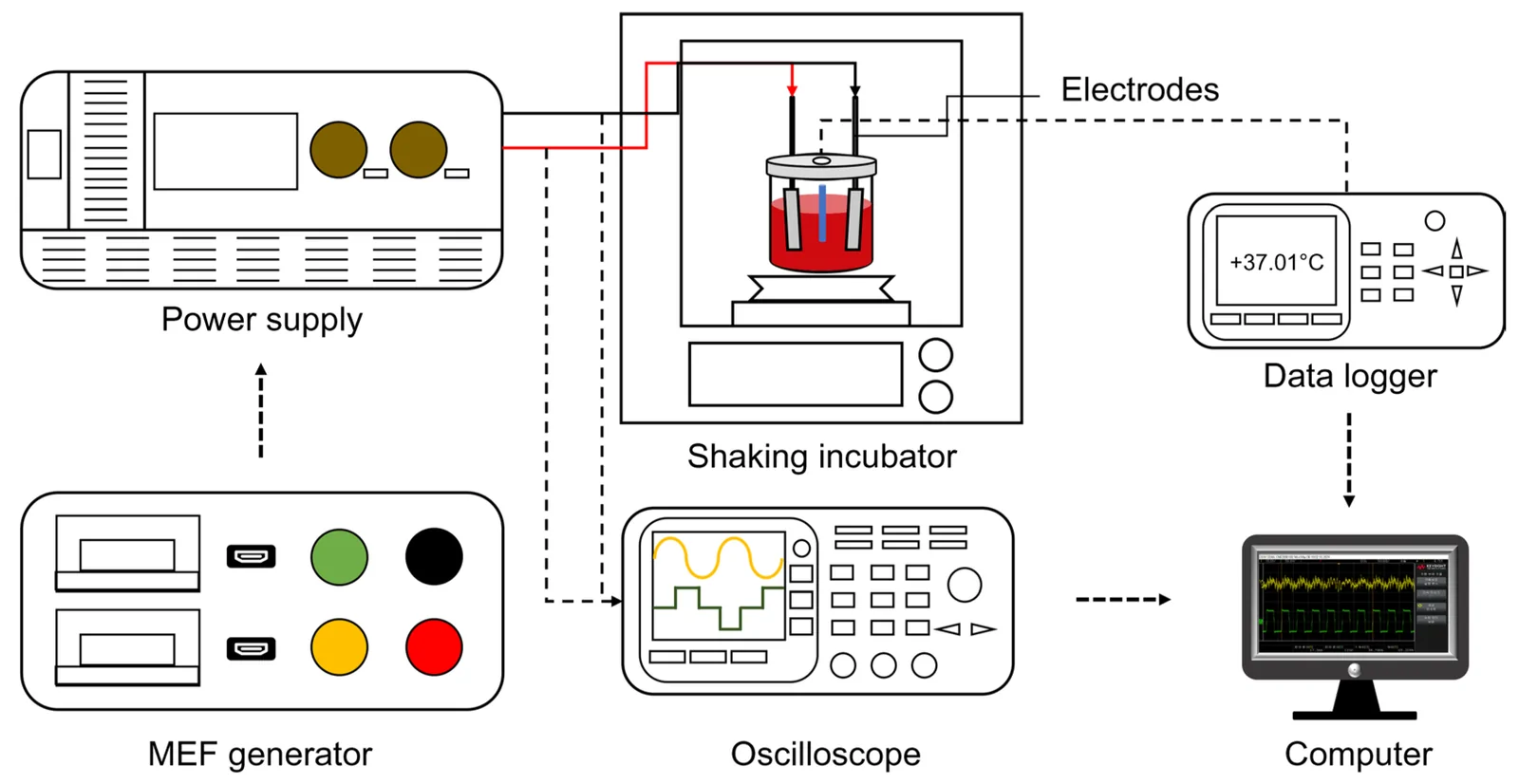
Schematic illustration of the moderate electric field (MEF)-assisted fermentation system. Electric pulses generated by the MEF signal generator were applied to the fermentation medium contained in a baffled reactor equipped with platinum–titanium (Pt–Ti) electrodes. The applied voltage and current were monitored in real time using a differential probe and a current probe connected to an oscilloscope. Temperature was measured using a type-K thermocouple connected to a data logger.

As shown in Figure 1, a baffled flat-bottom beaker reactor containing 150 mL of *Haematococcus*-based medium was placed in a shaking incubator operating at 160 rpm. Fixed Pt–Ti plate electrodes (40 mm × 60 mm × 1 mm) were installed in the reactor with an electrode gap of 5 cm and connected to the MEF generator. A differential probe (DP-25 oscilloscope probe, Pintek Electronics Co., Ltd., New Taipei City, Taiwan) was connected to the MEF generator to monitor the applied voltage, and a current probe (PT-2710, Pintek Electronics Co., Ltd.) was connected to the electrode wire attached to the Pt–Ti electrode to measure the current. To monitor the electrical signals in real-time, an oscilloscope (DSOX1204A, Keysight Technologies Inc., Santa Rosa, CA, USA) was connected to the current and differential probes.

For temperature monitoring, one end of a type K thermocouple was connected to a data acquisition system (DAQ970A, Keysight Technologies Inc., Santa Rosa, CA, USA), whereas the other end was positioned at the cold point of the suspension to continuously record temperature changes during MEF-assisted fermentation. The temperature was maintained at 37 ± 02 °C during treatment to minimize thermal effects. To prevent short circuits, the thermocouple and electrodes were positioned to avoid direct contact.

### 2.5. Effect of Enhancement Strategies of γ-PGA Biosynthesis in an Optimal Medium

#### 2.5.1. Effect of Membrane-Active Agents of γ-PGA Biosynthesis in the Optimized Medium

To evaluate the effects of membrane permeability and metabolite transport-modulating agents on γ-PGA biosynthesis, dimethyl sulfoxide (DMSO), Tween 80, and glycerol were supplemented into the optimized medium. The concentrations of each additive were determined based on the optimal conditions reported by [17] (DMSO: 0.1%; Tween 80: 0.3%; glycerol: 2.0%), and all additives were introduced at the initial stage of fermentation (Table S3).

#### 2.5.2. Physicochemical Stresses Affecting γ-PGA Biosynthesis in the Optimized Medium

To evaluate the effects of physicochemical stresses (KCl, heat, and pH) under the optimal medium conditions derived from RSM-BBD, experiments were conducted following a modified version of the method described by [18].

For KCl stress, KCl was added to the fermentation medium at the beginning of cultivation to achieve a final concentration of 3%. For heat shock, the *B. subtilis* HA inoculum was treated at 55 °C for 15 min and immediately cooled to 37 °C prior to use. In addition, to assess the effect of heat shock during fermentation, a thermal treatment at 50 °C for 15 min was applied at the initial stage of cultivation.

Alkaline stress was induced by adjusting the initial pH of the medium to 8.5 using 2 mol/L NaOH prior to fermentation.

### 2.6. Physicochemical Analysis of Conventional and MEF-Assisted Fermentation Products

#### 2.6.1. Quantitative Analysis of Mucilage Produced During Fermentation

To measure the mucilage yield, a 10 g sample of the fermented broth was mixed with 10 g of distilled water and vortexed. The resulting suspension was then centrifuged at 3134× *g* for 30 min, after which the supernatant was collected. The recovered supernatant was subsequently added to 40 g of isopropyl alcohol that had been stored at −20 °C in Erlenmeyer flasks to precipitate the mucilage. The aggregated polymer was retrieved using forceps and dried in a 60 °C dry oven for 24 h. The dry mass of the mucilage was then determined and used in Equation (1) to calculate its content:

(1) $$Mucilage\;yield\left(\%\right)=\frac{W_{d}}{W_{s}}\times 100\;(\%)$$

#### 2.6.2. γ-PGA Contents in Mucilage Fraction

To determine the γ-PGA content in the mucilage, the method described by [19] was followed with slight modifications. The mucilage fraction recovered from the fermented medium was washed three times with deionized water and dissolved in distilled water. The solution was then dialyzed against distilled water using a dialysis membrane with a molecular weight cut-off of 12–14 kDa for 24 h to remove low-molecular-weight compounds, including free glutamate and residual medium-derived glutamate. After dialysis, the mucilage fraction was lyophilized and used for γ-PGA content analysis.

The lyophilized mucilage fraction was hydrolyzed with 6 M HCl at 105 °C for 24 h. After hydrolysis, the reaction mixtures were neutralized using 6 M NaOH. The neutralized hydrolysates were diluted with the mobile phase and filtered through a 0.22 μm membrane filter prior to high-performance liquid chromatography (HPLC) analysis to reduce matrix effects caused by salts generated during neutralization and to remove insoluble particles.

The concentration of glutamate in both unhydrolyzed and hydrolyzed mucilage fraction samples was quantified using an HPLC system (Ultimate 3000, Thermo Fisher Scientific, Waltham, MA, USA) equipped with an Acclaim^TM^ 120 C18 column (4.6 mm × 250 mm) (Thermo Fisher Scientific, Waltham, MA, USA). The mobile phase consisted of 10% methanol (*v*/*v*, pH 2.5) and was delivered at a flow rate of 0.4 mL/min, with detection performed using a UV-Vis detector (Thermo Fisher Scientific, Waltham, MA, USA).

The γ-PGA content was estimated from the increase in glutamate concentration following acid hydrolysis and was calculated by subtracting the glutamate concentration of the unhydrolyzed sample from that of the hydrolyzed sample. This calculation was used to account, at least in part, for free glutamate present before hydrolysis. Because acid hydrolysis may also release glutamate from protein- or peptide-derived components, the calculated value was interpreted as an estimated γ-PGA content in the recovered mucilage fraction rather than an absolute direct quantification of purified γ-PGA. The content was calculated using the following equation (Equation (2)).

(2) $$\gamma \text{-}PGA\left(\%,w/v\right)=\frac{\left(C_{hydrolyzed}-C_{unhydrolyzed}\right)}{10}\times \frac{147}{129}$$

#### 2.6.3. L-Glutamate Contents in Fermented Medium

Residual glutamate concentrations after fermentation were quantified using the L-glutamic acid assay kit (Megazyme, Bray, Ireland) based on a microplate enzymatic method. The analysis was performed using a single-point standard; for each batch of samples, the calibration curve was prepared and analyzed concurrently with the samples using the same reagent lot. For each well, 200 µL of distilled water (blank), sample solution, or standard solution was mixed with solution 1 (buffer, 50 µL), solution 2 (NAD+/INT, 20 µL), and solution 3 (diaphorase, 5 µL). After incubation for 2 min, the initial absorbance (A_1_) was measured. The reaction was then initiated by the addition of solution 4 (glutamate dehydrogenase, 5 µL), followed by incubation at 25 °C for 10 min, after which the final absorbance (A_2_) was recorded.

L-glutamate concentrations were calculated according to the manufacturer’s equation (Equation (3)) and expressed in g/L based on the ratio of ΔA between the sample and the standard, multiplied by the dilution factor.

(3) $$L\text{-}glutamate\left(\%,w/v\right)=\frac{(\frac{∆A_{sample}}{∆A_{standard}})\times (\frac{g}{L}standard)\times F}{10}$$

#### 2.6.4. Calculation of Mucilage Yield Based on Substrate Consumption

The consumption of glucose and L-glutamate during fermentation was determined from the difference between their initial and residual contents in the culture broth. Reducing sugar content was expressed as an equivalent glucose content. Substrate consumption was calculated according to equations (Equations (4) and (5)):

(4) $$Glucose\;consumption\left(g/L\right)=G_{0}-G_{48}$$

(5) $$L\text{-}glutamate\;consumption\left(g/L\right)=L_{0}-L_{48}$$

where G_0_ and G_t_ denote the glucose contents at the onset of fermentation and at 48 h, respectively, and L_0_ and L_t_ denote the corresponding L-glutamate contents.

The overall mucilage yield based on substrate consumption (Y_P/S_) was defined as the amount of mucilage produced per unit mass of glucose and L-glutamate consumed, as was calculated using Equation (6):

(6) $$Y_{P/S}\left(g/L\right)=\frac{M_{48}-M_{0}}{\left(G_{0}-G_{48}\right)+(L_{0}-L_{48})}$$

where M_0_ and M_t_ represent the mucilage content at the onset of fermentation and at 48 h. Prior to calculation, the contents of mucilage, glucose, and L-glutamate were converted to a common unit (g/L). The resulting values of Y_P/S_ were expressed as grams of mucilage produced per gram of total substrate consumed (g/g). Yields were calculated independently for each biological replicate of the fermentation experiment.

#### 2.6.5. Measurement of Viable Bacterial Counts and Cell Growth Kinetics

Microbial growth was monitored during 48 h fermentation period by collecting aliquots at predetermined time intervals. For viable bacterial counts, 100 µL of each culture sample was serially diluted in 900 µL of sterile 0.85% NaCl solution to obtain dilutions. Subsequently, 20 µL of each dilution was spread onto MRS agar plates and incubated at 37 °C for 24 h. Viable cell counts were expressed as log CFU/mL.

In addition to viable cell enumeration, culture growth was by measuring the optical density at 600 nm (OD_600_) using EPOCH2 microplate reader (BioTek Instruments Inc., Winooski, VT, USA). The temporal profiles of OD_600_ were subsequently subjected to nonlinear regression against the Gompertz equation in order to derive descriptive growth-kinetic parameters. The model adopted the following equation (Equation (7))

(7) $$OD(t)=Y_{M}\cdot exp[-\exp\left(-K\left(t-t_{0}\right)\right)]$$

where OD(*t*) denotes the optical density at time *t*, *Y_M_* corresponds to the asymptotic (maximum) biomass expressed as OD_600_, K is the specific growth rate constant, and *t_0_* represents the inflection point marking the transition from the lag phase to exponential growth. Curve fitting and parameter estimation were carried out in GraphPad Prism (version 10.4.2; GraphPad Software, San Diego, CA, USA).

#### 2.6.6. Measurement of Glucose Consumption

Consumption of reducing sugar in the *H. pluvialis*-based broth was quantified by the 3,5-dinitrosalicylic acid (DNS) assay, employing D-glucose as the reference standard. Broth samples were first clarified by centrifugation (16,200× *g*, 15 min), after which the resulting supernatants were appropriately diluted. 1 mL of aliquot of each diluted supernatant was reacted with 3 mL of DNS reagent, and the mixture was heated in a boiling water bath (100 °C) for 5 min to develop the chromophore. The reaction was subsequently allowed to cool in the dark at 25 °C for 40 min, and the absorbance of the resulting solution was recorded at 550 nm.

### 2.7. Statistical Analysis

All fermentation experiments were performed in biological triplicate (n = 3), with five replicates at the central points of the Box–Behnken designs (BBD) to ensure model reproducibility. Results are expressed as the mean ± standard deviation (SD). Statistical analyses and graphs were generated with GraphPad Prism (version 10.4.2; GraphPad Software Inc., San Diego, CA, USA), and RSM design, modeling, and numerical optimization were performed with Design-Expert (version 23.0; Stat-Ease Inc., Minneapolis, MN, USA). Multiple conditions and fermentation time points were compared by one-way ANOVA followed by Tukey’s post hoc test, whereas pairwise comparisons between conventional (CF) and MEF-assisted fermentation (EF), including L-glutamate consumption, mucilage production, and substrate yield (Y_P/S_), were assessed by a two-tailed paired *t*-test. For BBD optimization of both medium composition and MEF operating conditions (with frequency on a logarithmic scale), second-order polynomial regression models were fitted, and their adequacy was evaluated by ANOVA, R_2_, adjusted R_2_, predicted R_2_, and the lack-of-fit test; the optimized conditions were then experimentally validated. Microbial growth kinetics were analyzed by nonlinear regression using the Gompertz equation. Statistical significance was defined at *p* < 0.05.

## 3. Results

### 3.1. Single-Factor Analysis for Optimization of Medium Composition Under Conventional Fermentation

**Figure 2:**
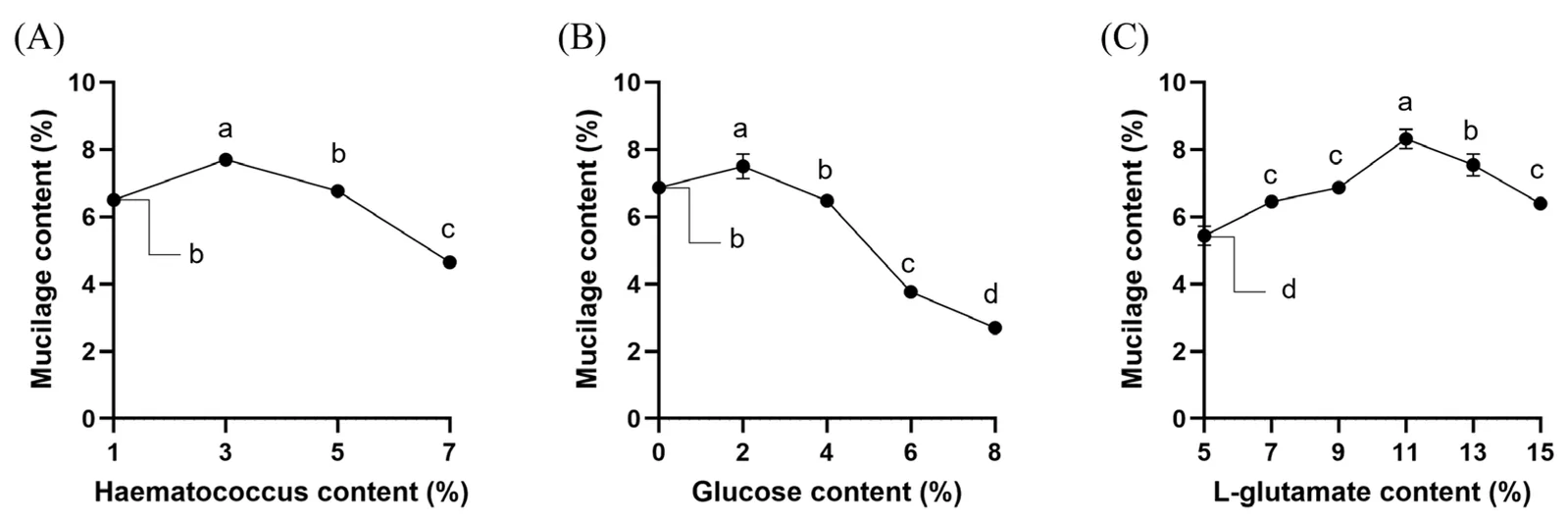
Mucilage yield in relation to *Bacillus subtilis*-driven biochemical modifications in the fermentation medium: (**A**) *Haematococcus pluvialis* content (%), (**B**) glucose content (%), and (**C**) L-glutamate content (%). ^a–d^: Statistically significant differences among substrate contents (*p* < 0.05).

Before optimizing the culture medium composition for *Haematococcus*-based fermentation using RSM-BBD, a single-factor analysis was conducted to determine the effective ranges of the three dependent variables (*H. pluvialis*, A; glucose, B; and L-glutamate, C). Through the comparison of the mucilage produced after 48 h of fermentation under varying concentrations of each medium component, the minimum, middle, and maximum levels for the subsequent RSM were established. *H. pluvialis* showed the highest mucilage yield at 3% (7.70%) (Figure 2A). Glucose exhibited its maximum effect at 2%, producing 7.50% mucilage (Figure 2B). Similarly, MSG achieved the highest production of mucilage at 11% (8.32%); however, mucilage production decreased to 6.40% at 15% MSG concentration (Figure 2C). Sodium derived from by *H. pluvialis* and MSG may function either as an osmotic stress factor or as a precursor/ionic regulator, promoting or inhibiting biopolymer synthesis depending on its concentration [20]. Glucose is known to induce CshA acetylation in *Bacillus* spp., enhancing the activity of sigma factors (SigX and SigM) that regulate the secretion and synthesis of EPS and extracellular enzymes [21]. Furthermore, the observed decrease in mucilage production at higher glutamate concentrations is consistent with previous reports indicating substrate inhibition when excessive glutamate is supplied to γ-PGA-producing microorganisms [22]. Based on these findings, the operational ranges for the independent factors were defined as follows: A (1–5%), B (0–4%), and C (5–15%).

### 3.2. Optimization of the Conventional Fermentation Conditions

Seventeen experimental runs were conducted based on combinations of three independent variables (A, B, and C) and one response variable (Y), and the mucilage yield varied depending on the composition of the fermentation medium (Table S1). The maximum yield (9.36 ± 0.10%) was obtained with a medium containing *H. pluvialis* 1%, glucose 4%, and MSG 10%, whereas the minimum yield (3.71 ± 0.36%) was observed in the medium composed of *H. pluvialis* 1%, glucose 2%, and MSG 5%.

Based on the results presented in Table S1, analysis of variance was performed to evaluate the statistical significance of the quadratic regression model (Table 1). The model exhibited a high level of significance, with an F-value of 35.57 and *p* < 0.0001, indicating that the selected independent variables effectively explained the variability in the response [23,24].

The lack-of-fit test, which evaluates whether the model adequately describes the experimental data relative to the pure error, showed an F-value of 4.86 and a *p*-value of 0.0803 (*p* > 0.05). This suggests that the regression model appropriately fits the experimental data [25].

The analysis of individual factors revealed that glucose and MSG significantly influenced mucilage production (*p* < 0.05), with MSG being the most dominant factor (F = 196.29, *p* < 0.0001). In addition, the significance of the quadratic term C^2^ indicates that mucilage production showed a nonlinear response to factor C, indicating the presence of an optimal condition for this variable.

**Figure 3:**
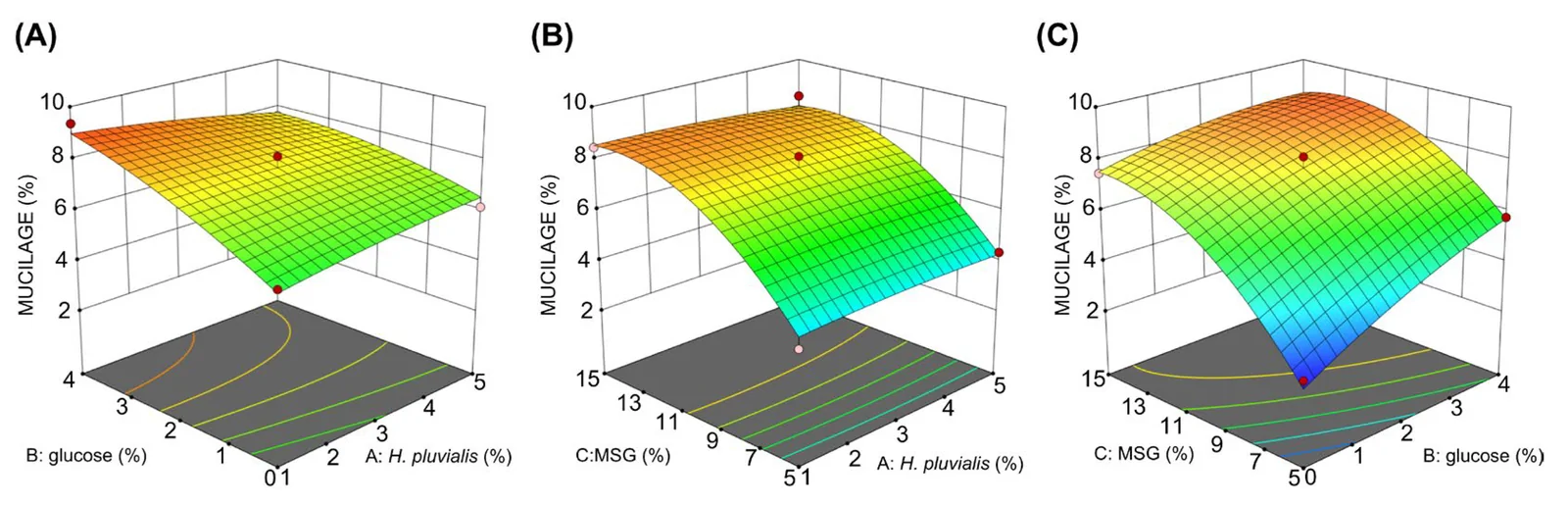
Three-dimensional response surface plots illustrating the interactive effects of (**A**) *Haematococcus pluvialis* and glucose (%), (**B**) *H. pluvialis* and monosodium glutamate (MSG, %), and (**C**) glucose and MSG (%) on mucilage production under conventional fermentation. The color represents the crude γ-polyglutamic acid (γ-PGA) yield. Red indicates a high concentration, and blue indicates a low concentration.

Regarding the interaction effects, the AB and BC terms were statistically significant, indicating that mucilage production was influenced by individual medium components and their combined effects. These interactions were further examined using 3D response surface plots (Figure 3A–C), which visually represent the behavior of the fitted regression model. The regression analysis showed a high coefficient of determination (R^2^ = 0.9786), whereas the adjusted R^2^ value of 0.9511 confirmed the robustness of the model in explaining the response variability without undue influence from unnecessary variables. Furthermore, the predicted R^2^ value (0.7241) indicated reasonable agreement between the experimental and predicted values.

To validate the predictive accuracy of the optimal conditions derived from the model equation, experiments were conducted using the proposed solution. The predicted mucilage production obtained from the solution was 8.54%, whereas the experimental value was 8.60 ± 0.25%, indicating the adequacy and robustness of the developed model (Table 2).

### 3.3. Temperature Changes in the Medium According to Frequency, Current, and Duty Cycle

To verify the intrinsic effects of electrical stimulation during fermentation, it is essential to eliminate any thermal effects. Most food matrices contain a large amount of free water and dissolved salts, resulting in high electrical conductivity. When exposed to an electric field, these ions migrate toward electrodes of the opposite charge, and ionic collisions that occur during this process can lead to an increase in the internal temperature of the food matrix [26,27].

**Figure 4:**
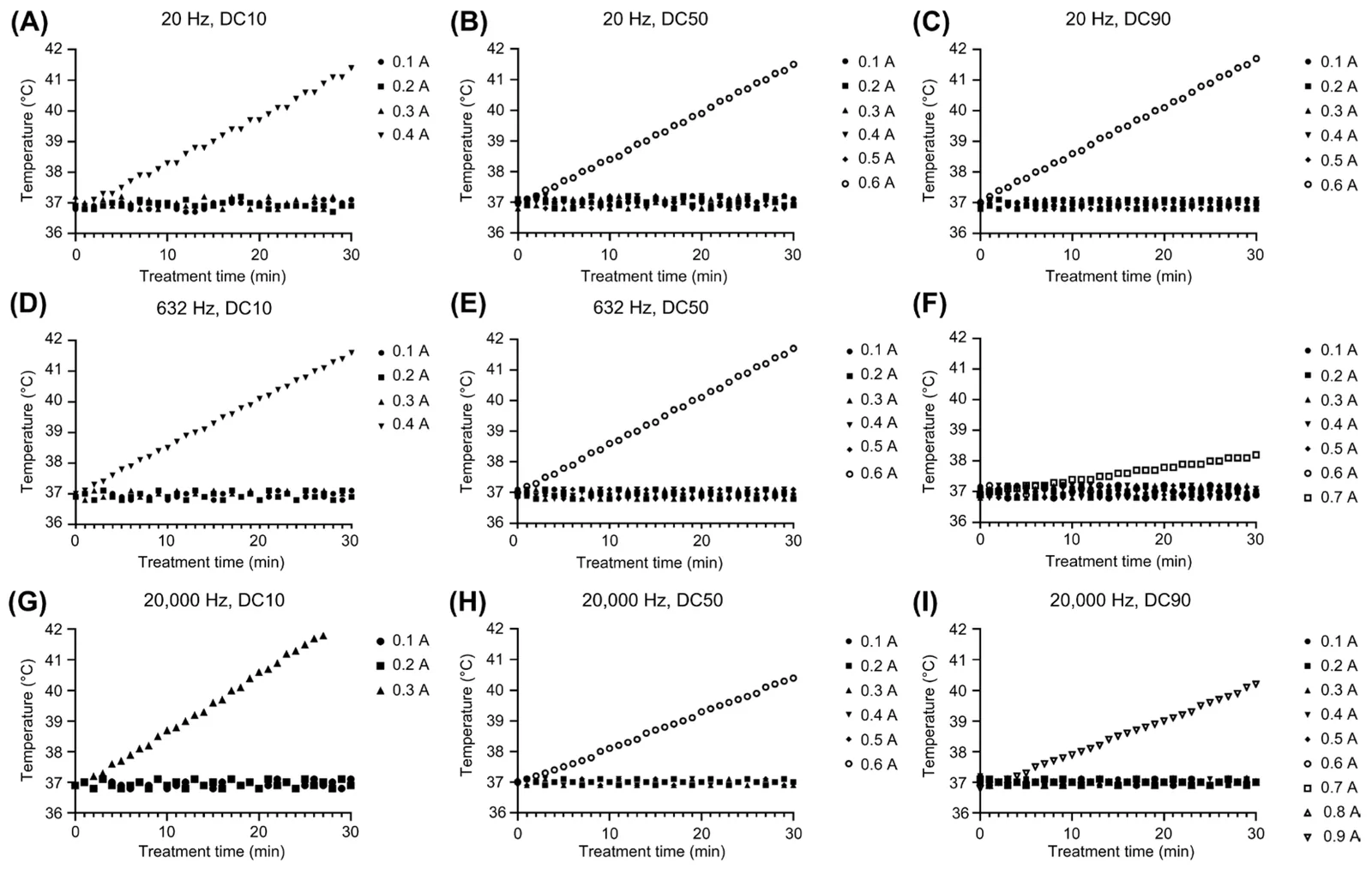
Temperature increase behavior under electrical stimulation at different frequencies, duty cycles, and current intensities. Joule heating was monitored as a function of the applied current at low frequency (20 Hz) with duty cycles of 10%, 50%, and 90% (**A**–**C**), mid frequency (632 Hz) (**D**–**F**), and high frequency (20,000 Hz) (**G**–**I**). Ohmic heating varied depending on the frequency and duty cycle, indicating frequency- and duty cycle-dependent electrothermal responses during electrical treatment.

At a frequency of 20 Hz, an increase in temperature was observed at 0.4 A for a 10% duty cycle, 0.6 A for 50%, and 0.6 A for a 90% duty cycle (Figure 4A–C). Similarly, at a mid-frequency (632 Hz), ohmic heating occurred at 0.4, 0.6, and 0.7 A for duty cycles of 10%, 50%, and 90%, respectively (Figure 4D–F). In the case of high frequency (20 kHz), the temperature of the medium was increased at 0.3 A, 0.6 A, and 0.9 A. This phenomenon can be attributed to the enhanced charge migration and electrolyte release induced by structural changes in cells at lower frequencies, which increases electrical conductivity and consequently improves heating efficiency [28].

In addition, an increase in the duty cycle reduces heat generation because the electrical double layer at the electrode-electrolyte interface does not discharge sufficiently, decreasing the fraction of electrical energy converted into thermal energy [29].

However, when defining the experimental conditions for optimization using RSM, it is critical to minimize the possibility of temperature elevation. Therefore, the experimental parameters were set within the following ranges: frequency of 20–20,000 Hz, duty cycle of 10–90%, and current intensity of 0.1–0.3 A.

### 3.4. Optimization of the MEF-Fermentation Conditions

To evaluate the effects of fermentation process variables on mucilage production, RSM based on RSM-BBD was used. The results indicate that the experimental data were well fitted to a quadratic model. Statistical validation by analysis of variance showed that the overall regression model was highly significant, with an F-value of 17.79 and a *p*-value of 0.0005 (*p* < 0.001), confirming that the independent variables effectively explained the variability in mucilage production (Table 3).

Analysis of the main effects revealed that factors A (frequency) and B (current value) significantly influenced mucilage production. In contrast, the linear term of factor C (duty cycle) was not significant (*p* = 0.9347); however, its quadratic term (C^2^, *p* = 0.0295) was statistically significant, indicating a nonlinear effect of the duty cycle and suggesting the presence of an optimal operating range. Similarly, the quadratic term of the current value (B^2^, *p* = 0.0288) was also significant, demonstrating that the effects of current value on mucilage production followed a curved pattern, rather than a purely linear response pattern.

**Figure 5:**
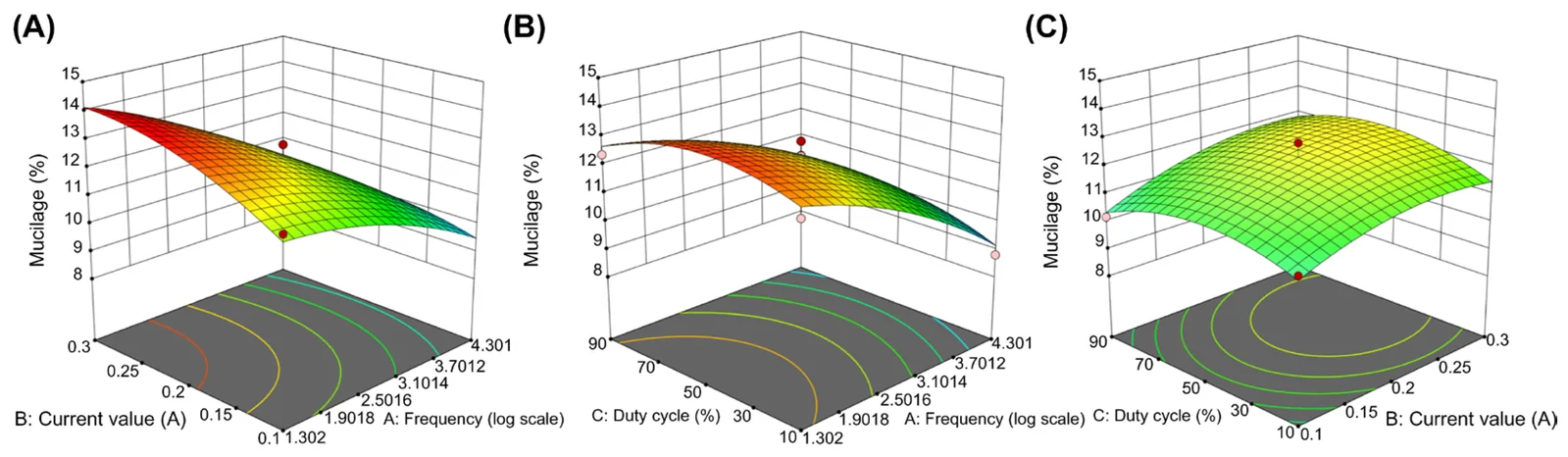
Three-dimensional response surface plots illustrating the interactive effects of (**A**) log frequency and current intensity, (**B**) log frequency and duty cycle, and (**C**) current intensity and duty cycle on mucilage production under conventional fermentation. The color represents the crude γ-PGA yield. Red indicates a high concentration, and blue indicates a low concentration.

Figure 5A–C illustrates the interaction effects among factors A, B, and C on mucilage production. The mucilage yield decreased with increasing frequency, whereas an increase in the current value enhanced mucilage production. The maximum production region was observed under conditions of low frequency combined with a high electric field strength. In alternating electric fields applied in a square-wave form, the intensity of electropermeabilization increases as the alternating current frequency ranges from 1 to 100 kHz. This is because insufficient membrane charging time prevents the induced transmembrane voltage from reaching the critical threshold required for electroporation. Although increasing the electric field strength can enhance electroporation [30], excessive treatment may lead to bacterial inactivation rather than controlled permeabilization [31,32].

The 3D response surface plot analysis demonstrated that the interaction between frequency and current value, together with the nonlinear effect of the duty cycle, plays a crucial role in optimizing mucilage production under the studied fermentation conditions. Accordingly, numerical optimization identified the optimal MEF-fermentation conditions as a frequency of 85.2 Hz, current of 0.29 A, and duty cycle of 40.90%, yielding a predicted mucilage production of 14.58%. This value was in close agreement with the experimental result (13.59 ± 0.18%), confirming the validity of the developed model (Table 4).

### 3.5. Growth Kinetics of B. subtilis in Conventional and MEF-Assisted Fermentation

**Figure 6:**
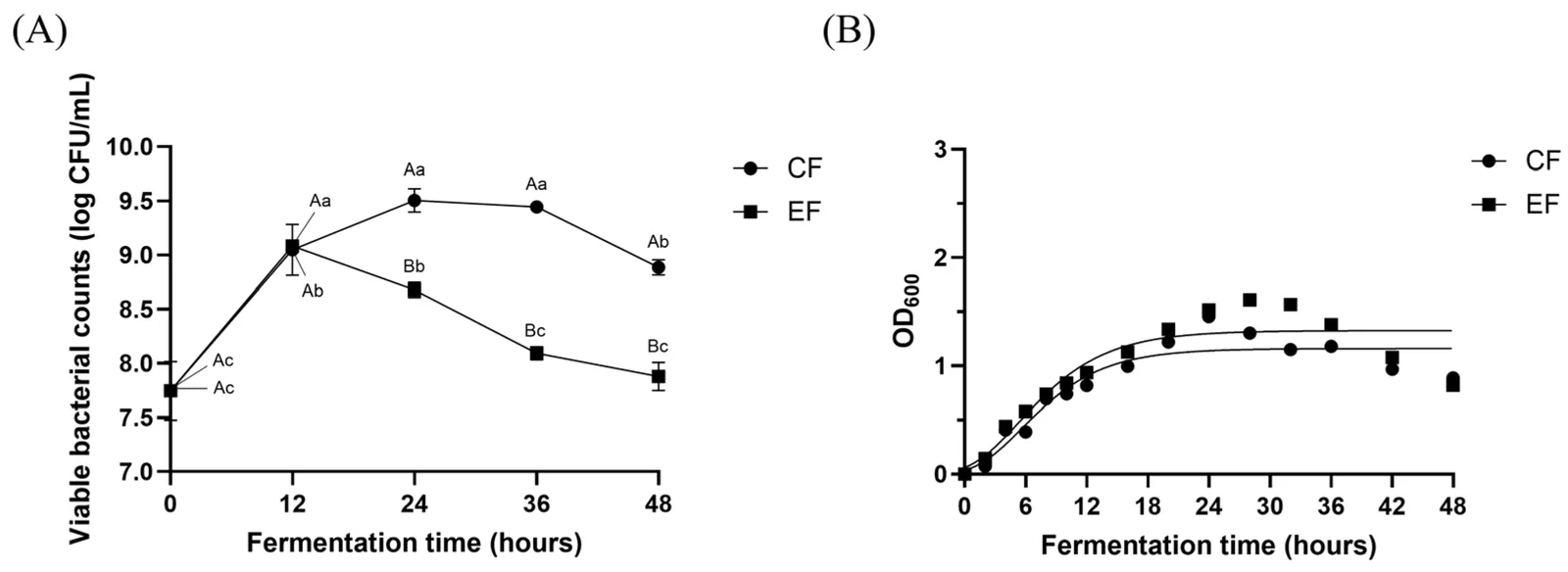
(**A**) Viable bacterial counts and (**B**) Growth kinetics of *Bacillus subtilis* during conventional fermentation (CF) and MEF-assisted fermentation (EF). Cell growth was monitored by measuring optical density at 600 nm (OD_600_) over 48 h of fermentation. Circles and squares represent the experimental data for CF and EF, respectively, and solid lines indicate the fitted Gompertz growth curves. ^A,B^: statistically significant differences among conditions (*p* < 0.05). ^a–c^: statistically significant differences among fermentation durations (*p* < 0.05). Data are expressed as mean ± standard deviation (n = 3). Data are expressed as mean ± standard deviation (n = 3).

As shown in Figure 6, the growth characteristics of *Bacillus subtilis* under conventional fermentation (CF) and moderate electric field-assisted fermentation (EF) were evaluated using both viable bacterial counts (Figure 6A) and optical density at 600 nm (OD_600_) over 48 h (Figure 6B). Viable bacterial counts increased rapidly during the initial 12 h under both fermentation conditions. Under CF, the viable count increased from 7.747 ± 0.270 log CFU/mL at 0 h to 9.050 ± 0.235 log CFU/mL at 12 h and reached a maximum of 9.507 ± 0.107 log CFU/mL at 24 h. The viable count remained relatively high at 36 h (9.447 ± 0.031 log CFU/mL) and subsequently decreased to 8.887 ± 0.071 log CFU/mL at 48 h. In contrast, the EF group reached its maximum viable count earlier, recording 9.083 ± 0.038 log CFU/mL at 12 h, followed by a progressive decline to 8.680 ± 0.072, 8.093 ± 0.061, and 7.880 ± 0.131 log CFU/mL at 24, 36, and 48 h, respectively. The viable-count-based specific growth rates during the initial 0–12 h were comparable between CF and EF, at 0.250 ± 0.016 and 0.256 ± 0.057 h^−1^, respectively. These results indicate that EF did not substantially increase the early specific growth rate but induced an earlier transition from the growth phase to the stationary or decline phase.

The growth kinetics of *B. subtilis* under CF and EF were characterized by monitoring OD_600_ over 48 h and fitting the resulting data to a Gompertz growth model. The modified Gompertz equation has been widely accepted as a robust three-parameter model for describing microbial growth curves, offering statistically reliable estimation of the lag time, maximum specific growth rate, and asymptotic maximum population [33]. Under both conditions, OD_600_ increased rapidly during the early stage of fermentation. However, the EF group generally showed higher OD_600_ values than the CF group from 2 to 42 h. This early-stage acceleration of biomass accumulation is consistent with previous reports showing that moderate electric fields (MEF) can enhance microbial growth by increasing cell membrane permeability and improving nutrient uptake, without inducing lethal electroporation [34].

The maximum measured OD_600_ was 1.456 ± 0.009 at 24 h for CF and 1.610 ± 0.013 at 28 h for EF. EF group also maintained a relatively high OD_600_ until 32 h, indicating prolonged apparent biomass accumulation. Similar growth-promoting effects have been reported for lactic acid bacteria and yeasts subjected to mild pulsed or moderate electric fields, in which the treatment shortened the lag phase and elevated the maximum viable cell density [35,36]. Gompertz model analysis yielded higher asymptotic maximum OD_600_ values for EF than for CF, with values of 1.327 and 1.160, respectively. Their 95% confidence intervals did not overlap, indicating a statistically meaningful enhancement of the carrying capacity by EF, in agreement with recent findings that sublethal electric fields primarily elevate the plateau biomass rather than modify the exponential slope [37].

The growth rate constants were similar between CF and EF (0.2202 vs. 0.2078) with overlapping confidence intervals, and the Gompertz model fit was acceptable (R^2^ = 0.8925 for CF, 0.8442 for EF). After 32 h, OD_600_ decreased in both conditions, with a more pronounced decline in the EF group. This is likely attributable to nutrient depletion and stationary-phase autolysis, combined with mass-transfer limitations caused by elevated γ-PGA accumulation, which markedly increases broth viscosity and restricts oxygen transfer [38,39]. Overall, EF did not alter the intrinsic growth rate but increased the maximum culture turbidity and prolonged the high-cell-density phase.

### 3.6. Reducing Sugar Consumption in Conventional and MEF-Assisted Fermentation

**Figure 7:**
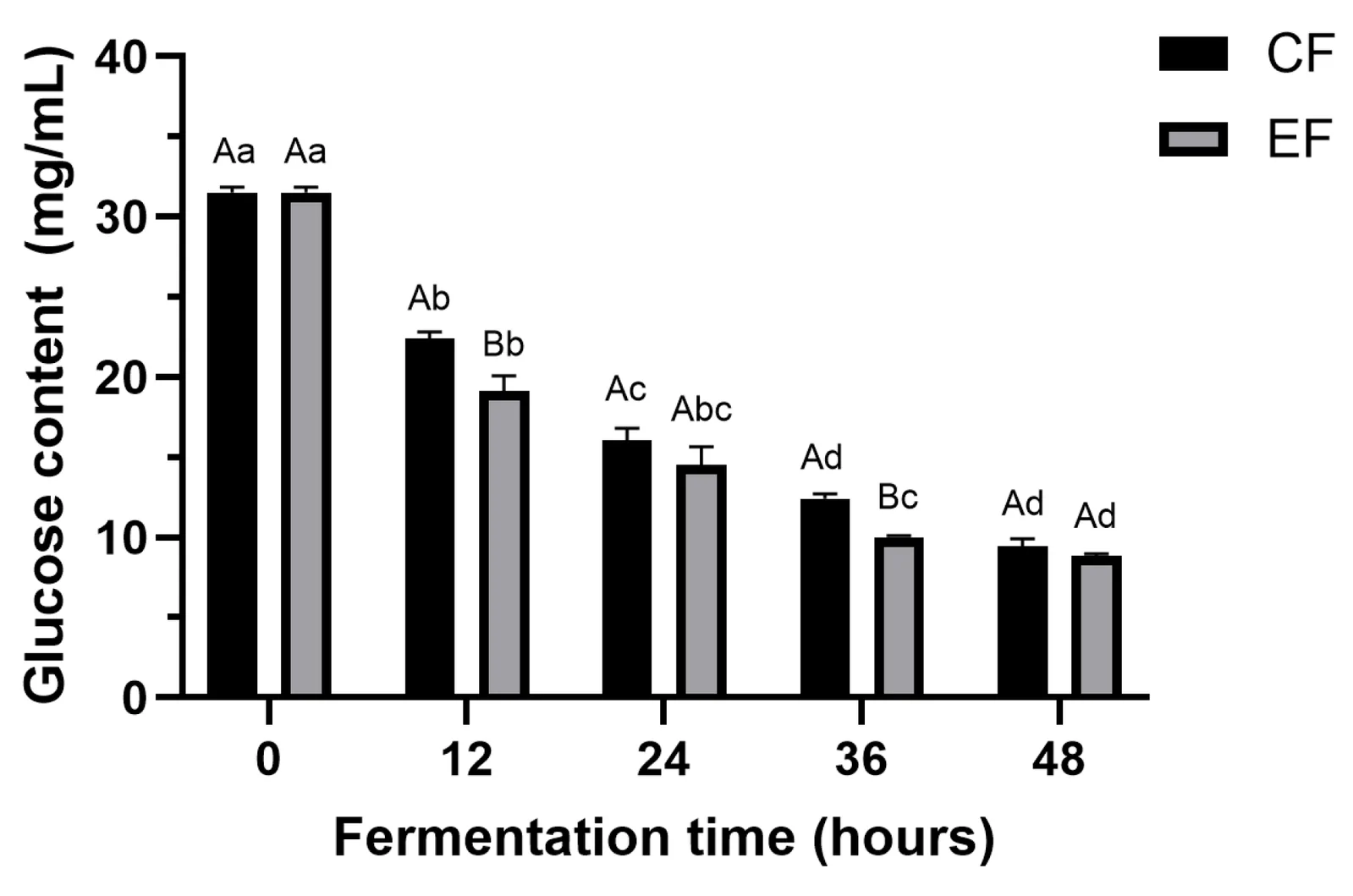
Glucose content during fermentation during conventional fermentation (CF) and MEF-assisted fermentation (EF). ^A,B^: statistically significant differences among conditions (*p* < 0.05). ^a–d^: statistically significant differences among fermentation durations (*p* < 0.05). Data are expressed as mean ± standard deviation (n = 3).

The reducing sugar content decreased continuously during 48 h of fermentation in both CF and EF (Figure 7). EF showed significantly lower reducing sugar levels than CF at 12 and 36 h (*p* < 0.05), whereas no significant differences were observed at 24 and 48 h. Final reducing sugar consumption was similar between CF and EF, reaching 70.06% and 71.96%, respectively. These results indicate that MEF accelerated sugar utilization during the early and intermediate fermentation stages rather than increasing the overall extent of consumption. The enhanced substrate utilization under EF may have supported greater microbial growth and mucilage production by promoting carbohydrate transport and metabolic activity [36].

### 3.7. Mucilage Production in Conventional and MEF-Assisted Fermentation

**Figure 8:**
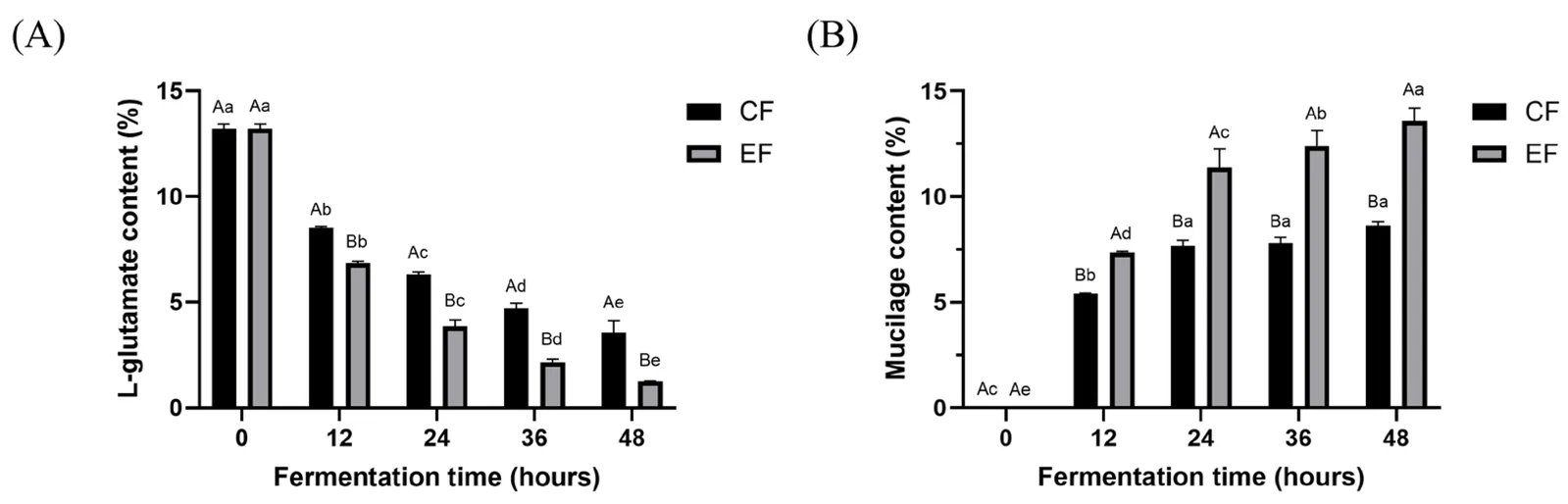
Changes in residual L-glutamate and mucilage (crude γ-PGA) content during fermentation during conventional fermentation (CF) and MEF-assisted fermentation (EF). (**A**) Residual L-glutamate content and (**B**) mucilage (crude γ-PGA) content production. ^A,B^: statistically significant differences among conditions (*p* < 0.05). ^a–e^: statistically significant differences among fermentation durations (*p* < 0.05). Data are expressed as mean ± standard deviation (n = 3).

Residual L-glutamate decreased progressively, whereas mucilage accumulated under both conventional fermentation (CF) and moderate electric field-assisted fermentation (EF) (Figure 8). The initial L-glutamate content was 13.21 ± 0.22% and decreased to 3.57 ± 0.56% under CF and 1.25 ± 0.03% under EF after 48 h. Accordingly, L-glutamate consumption was significantly higher under EF than under CF (11.96 ± 0.21 vs. 9.64 ± 0.68 percentage points, respectively; *p* = 0.0201; Table 5). Furthermore, residual L-glutamate levels were significantly lower under EF from 12 h onward, indicating enhanced substrate utilization during fermentation.

Mucilage production increased from 5.40 ± 0.04% and 7.36 ± 0.04% at 12 h to 8.62 ± 0.19% and 13.58 ± 0.62% at 48 h under CF and EF, respectively. Thus, EF increased the final mucilage content by approximately 57.5% compared with CF (*p* = 0.0029). Under CF, mucilage accumulation largely plateaued after 24 h, whereas it continued until 48 h under EF, indicating that MEF prolonged the effective production period into the later stages of fermentation.

The overall substrate yield (Y_P/S_), calculated based on the total consumption of glucose and L-glutamate, was also significantly higher under EF than under CF (0.955 ± 0.059 vs. 0.729 ± 0.033 g mucilage/g total substrate consumed; *p* = 0.0089; Table 5). These results indicate that MEF enhanced not only substrate utilization but also the conversion efficiency of the consumed substrates into mucilage. This improvement may be associated with MEF-induced increases in cell membrane permeability and enhanced substrate transport [40]. However, because membrane properties and metabolic fluxes were not directly evaluated in the present study, further investigation is required to clarify the underlying mechanism. 

### 3.8. Comparison of Residual L-Glutamate, γ-PGA, and Mucilage Contents Under Different Stress Conditions

**Figure 9:**
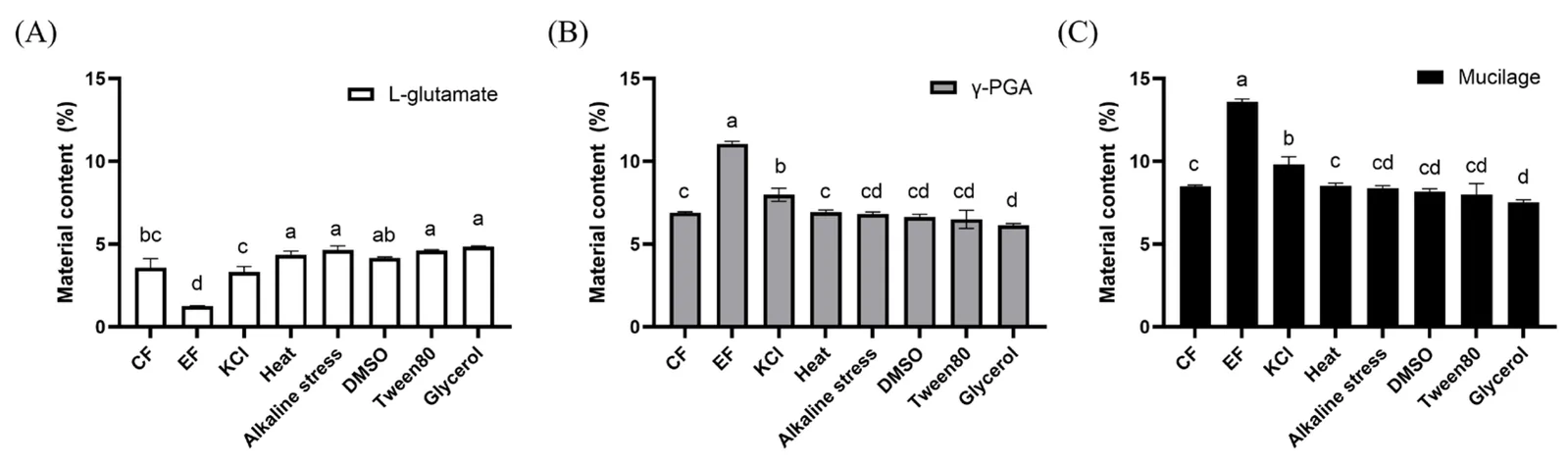
Effects of physicochemical stress (KCl, heat, alkaline stress) and membrane-active additives (dimethyl sulfoxide [DMSO], Tween 80, glycerol) on (**A**) L-glutamate content and (**B**) γ-polyglutamic acid (γ-PGA) production, and (**C**) mucilage content under optimized fermentation conditions. Data are expressed as mean ± standard deviation (n = 3). CF, conventional fermentation; EF, electro-fermentation. Different lowercase letters (^a–d^) indicate significant differences among treatments (*p* < 0.05).

To evaluate the effect of MEF-assisted fermentation on L-glutamate bioconversion, residual L-glutamate and γ-PGA contents, and mucilage yield were compared under CF, EF, membrane-active agents, and physicochemical stress conditions (Figure 9). As L-glutamate is the direct precursor of γ-PGA, which constitutes the major component of mucilage, these parameters collectively reflect substrate utilization and extracellular polymer production.

EF treatment resulted in the lowest residual L-glutamate content (1.25 ± 0.03%) (Figure 9A), indicating enhanced substrate consumption compared with CF (3.57 ± 0.56%) and other stress conditions. Correspondingly, EF treatment resulted the highest γ-PGA content (11.07 ± 0.16%) (Figure 9B) and mucilage yield (13.59 ± 0.18%) (Figure 9C), both of which were significantly higher than those of CF (6.97 ± 0.16% and 8.55 ± 0.20%, respectively). KCl treatment led to moderately increased γ-PGA content (7.99 ± 0.39%) and mucilage yield (9.81 ± 0.48%), consistent with previous reports indicating that K^+^ enhances γ-PGA biosynthesis via glutamate-related enzymes and *pgsB* expression [41]. In contrast, heat, alkaline stress, DMSO, Tween 80, and glycerol showed relatively minimal effects.

Although DMSO, Tween 80, and glycerol reportedly enhance γ-PGA biosynthesis, their effects are based on distinct metabolic and physiological mechanisms. Tween 80 and DMSO primarily lead to increased cell membrane permeability, facilitating extracellular substrate uptake and γ-PGA secretion, while glycerol promotes γ-PGA biosynthesis mainly by redirecting carbon flux from 2-oxoglutarate toward glutamate accumulation through suppression of 2-oxoglutarate dehydrogenase complex activity [42]. However, these additives did not enhance γ-PGA or mucilage production under the present *Haematococcus*-based fermentation conditions. This suggests that the expected membrane permeability enhancement or carbon flux redistribution was not effectively translated into γ-PGA biosynthesis, likely attributable to differences in *Bacillus* strain-specific responses, additive concentration, medium composition, or the presence of microalgal matrix.

These results indicate that EF markedly enhanced the conversion of L-glutamate into γ-PGA-rich mucilage. The simultaneous decrease in residual L-glutamate, as well as the increases in γ-PGA content and mucilage yield indicate enhanced metabolic flux toward extracellular polymer synthesis rather than a nonspecific stress response. Although conventional stress conditions showed limited or inconsistent responses, Electrical stimulation appears to act as a more effective and controllable stimulus for enhancing γ-PGA production in *B. subtilis* fermentation. Nevertheless, further enzymatic or metabolic analyses would be required to validate the regulatory mechanisms underlying MEF-induced γ-PGA biosynthesis.

The enhancement of γ-PGA production under MEF-assisted fermentation may be explained by the interaction between electrical stimulation and the membrane-associated nature of γ-PGA biosynthesis in *Bacillus subtilis*. γ-PGA synthesis requires the supply of L-/D-glutamate precursors, ATP-dependent polymerization, and extracellular transfer through the PgsBCA synthetase system [43]. Therefore, changes in cell-envelope permeability or membrane-associated transport can potentially influence both precursor availability and polymer secretion. Sublethal electric-field treatment has been reported to induce reversible changes in microbial membrane permeability and metabolic activity without necessarily causing irreversible cell damage [44]. In addition, electrofermentation has recently been reported to enhance γ-PGA production in *Bacillus subtilis* biofilms, supporting the possibility that electrical stimulation can modulate γ-PGA-associated biosynthesis. In this study, the optimized MEF condition increased γ-PGA-containing mucilage production and reduced residual glutamate compared with conventional fermentation, suggesting that the applied electric field may have improved the conversion of exogenous glutamate into extracellular γ-PGA. This interpretation aligns with [15], who showed that *B. subtilis* HA possesses a glutamate-responsive metabolic axis directing exogenous L-glutamate toward γ-PGA biosynthesis. The present findings suggest that MEF amplifies this pre-existing axis by enhancing transmembrane precursor transport, rather than inducing a nonspecific stress response. However, because transcriptomic, proteomic, membrane-potential, and intracellular ATP analyses were not performed, this mechanism should be regarded as a plausible interpretation rather than a direct demonstration. Future studies should examine MEF-induced changes in membrane permeability, intracellular energy/redox status, and the expression of γ-PGA synthesis- and degradation-related genes such as pgsBCA, racE, degU/degQ, pgdS, ggt, and cwlO.

## 4. Conclusions

MEF-assisted fermentation enhanced γ-PGA-rich mucilage production by *B. subtilis* HA in an *H. pluvialis*-based medium. Using RSM-BBD, the optimal formulation for CF was determined to be 2.91% *H. pluvialis*, 3.10% glucose, and 13.42% L-glutamate. Under these conditions, the experimental mucilage yield (8.60 ± 0.25%) closely matched the predicted value (8.54%), confirming the adequacy of the developed model.

To minimize thermal interference from ohmic heating, MEF parameters were optimized under controlled nonthermal conditions. The optimized MEF conditions were identified as 85.2 Hz, 0.29 A, and 40.90% duty cycle, resulting in an experimental mucilage yield of 13.59 ± 0.18%. Under the optimized MEF conditions, residual L-glutamate was reduced and γ-PGA content in the recovered mucilage fraction was increased, indicating that MEF-assisted fermentation promoted γ-PGA-rich mucilage formation.

Compared with other stress treatments, MEF showed a more pronounced enhancement effect under the present fermentation conditions. These findings suggest that MEF may serve as a controllable physical strategy for improving γ-PGA-associated EPS production in *B. subtilis* fermentation using microalgal biomass-based media. Further studies are required to clarify the underlying cellular and metabolic mechanisms and to evaluate the reproducibility, scale-up feasibility, energy efficiency, and product quality of MEF-assisted fermentation.

## Figures and Tables

**Table 1:** Analysis of variance and adequacy of the quadratic model for medium composition.

Source	Sum of Squares	Degrees of Freedom	Mean Square	F-Value	*p*-Value
Model	53.97	9	6.00	35.57	<0.0001
A	0.08	1	0.08	0.47	0.5131
B	10.06	1	10.06	59.66	0.0001
C	33.09	1	33.09	196.29	<0.0001
AB	1.04	1	1.04	6.17	0.0419
AC	0.10	1	0.10	0.57	0.4749
BC	1.36	1	1.36	8.05	0.0251
A^2^	0.06	1	0.06	0.33	0.5843
B^2^	0.44	1	0.44	2.59	0.1513
C^2^	7.42	1	7.42	44.00	0.0003
Residual	1.19	7	0.17		
Lack of Fit	0.93	3	0.3087	4.86	0.0803
Pure Error	0.24	4	0.06		
Cor Total	55.15	16			
R^2^	0.98				
Adjusted R^2^	0.95				
Predicted R^2^	0.72				
Regression equation			Y = −5.64119 + 0.53212 A + 1.84787 B + 1.63155 C − 0.12750 AB − 0.01550 AC − 0.05825 BC − 0.02869 A^2^ − 0.08056 B^2^ − 0.05309 C^2^		

Statistically significant, *p* < 0.05; statistically highly significant, *p* < 0.01; statistically very significant, *p* < 0.001; statistically extremely significant, *p* < 0.0001.

**Table 2:** Comparison of the predicted and experimental responses under optimum conditions for conventional fermentation.

Independent Variables	Optimum Conditions	
*Haematococcus pluvialis* (%)	2.91	
Glucose (%)	3.10	
MSG (%)	13.42	
Responses	Predicted value	Experimental value
Mucilage production (%)	8.54	8.60 ± 0.25

Data are expressed as mean ± standard deviation (n = 3).

**Table 3:** Analysis of variance and adequacy of the quadratic model for MEF operation.

Source	Sum of Squares	Degrees of Freedom	Mean Square	F-Value	*p*-Value
Model	48.17	9	5.35	19.14	0.0004
A	26.87	1	26.87	96.08	<0.0001
B	2.87	1	2.87	10.26	0.0150
C	0.0151	1	0.0151	0.0540	0.8230
AB	1.01	1	1.01	3.59	0.0998
AC	0.1726	1	0.1726	0.6173	0.4578
BC	0.1382	1	0.1382	0.4940	0.5048
A^2^	0.7701	1	0.7701	2.75	0.1410
B^2^	1.02	1	1.02	3.66	0.0974
C^2^	1.23	1	1.23	4.41	0.0739
Residual	1.96	7	0.2797		
Lack of Fit	1.68	4	0.4211	4.62	0.1195
Pure Error	0.2733	3	0.0911		
Cor Total	50.13	16			
R^2^	0.9609				
Adjusted R^2^	0.9107				
Predicted R^2^	0.6956				
Regression equation	Y = 12.33X − 1.72A + 0.5991B + 0.0395C − 0.5151AB + 0.1671AC + 0.1620BC − 0.5159A^2^ − 0.5248B^2^ − 0.7506C^2^				

Statistically significant, *p* < 0.05; statistically highly significant, *p* < 0.01; statistically very significant, *p* < 0.001; statistically extremely significant, *p* < 0.0001.

**Table 4:** Comparison of the predicted and experimental responses under optimum conditions for MEF-assisted fermentation.

Independent Variables	Optimum Conditions	
Frequency (Hz)	85.2	
Current value (A)	0.29	
Duty cycle (%)	40.90	
Responses	Predicted value	Experimental value
Mucilage production (%)	14.58	13.59 ± 0.18

Data are expressed as mean ± standard deviation (n = 3).

**Table 5:** L-glutamate consumption, mucilage production, and overall substrate conversion yield under conventional fermentation and MEF-assisted fermentation.

Parameter	CF	EF	*p*-Value
L-glutamate consumption during fermentation (%)	9.85 ± 0.56 ^b^	12.17 ± 0.03 ^a^	0.0201
Mucilage production during fermentation (%)	6.90 ± 0.06 ^b^	11.07 ± 0.16 ^a^	0.0029
Overall substrate yield *Y_P/S_* (g mucilage/total substrate)	0.57 ± 0.03 ^b^	0.77 ± 0.01 ^a^	0.0089

Values are expressed as mean ± standard deviation (n = 3). Different superscript letters (^a,b^) within the same row indicate significant differences between CF and MEF according to a two-tailed paired *t*-test (*p* < 0.05). CF, conventional fermentation; EF, MEF-assisted fermentation; Overall substrate yield (Y_P/S_) was calculated as the amount of mucilage produced per total amount of glucose and L-glutamate consumed during 48 h of fermentation.

## Data Availability

The original contributions presented in this study are included in the Article/Supplementary Materials. Further inquiries can be directed to the corresponding authors.

## Supplementary Materials

The following supporting information can be downloaded at: https://www.mdpi.com/article/10.3390/microorganisms14081732/s1, Table S1: Box–Behnken design scheme for medium compositions and results of mucilage production by *Bacillus subtilis* HA; Table S2: Box–Behnken design scheme for moderate electric field fermentation and results of mucilage production by *Bacillus subtilis* HA; Table S3: Components of fermentation media to evaluate membrane-active reagents.
