# Supplementary material for: Optimization of Poly-γ-Glutamic Acid Production by Moderate Electric Field-Assisted Fermentation in a Haematococcus pluvialis-Based Medium
Source: Microorganisms. 2026 Aug 7;14(8):1732. doi: 10.3390/microorganisms14081732 (PMC13515176; doi:10.3390/microorganisms14081732)
Supplement: Supplementary file 1 [file microorganisms-14-01732-s001.zip › microorganisms-4363419-supplementary.pdf]

Supplementary Material

Table S1. Box–Behnken design scheme for medium compositions and results of mucilage production by *Bacillus subtilis* HA.

Run	A: *Haematococcus pluvialis* (%)	B: glucose (%)	C: L-glutamate (%)	Y: Mucilage yield (%)
1	3 (0)	0 (−1)	15 (+1)	8.47 ± 0.28
2	5 (+1)	4 (+1)	10 (0)	7.62 ± 0.34
3	3 (0)	4 (+1)	5 (−1)	5.76 ± 0.09
4	5 (+1)	0 (−1)	10 (0)	6.15 ± 0.18
5	1 (−1)	2 (0)	15 (+1)	8.45 ± 0.36
6	3 (0)	2 (0)	10 (0)	6.62 ± 0.18
7	5 (+1)	2 (0)	5 (−1)	4.34 ± 0.32
8	3 (0)	4 (+1)	15 (+1)	8.30 ± 0.08
9	1 (−1)	2 (0)	5 (−1)	3.10 ± 0.36
10	3 (0)	0 (−1)	5 (−1)	3.71 ± 0.20
11	3 (0)	2 (0)	10 (0)	7.11 ± 0.69
12	3 (0)	2 (0)	10 (0)	8.13 ± 0.32
13	5 (+1)	2 (0)	15 (+1)	8.46 ± 0.56
14	1 (−1)	4 (+1)	10 (0)	7.57 ± 0.10
15	3 (0)	2 (0)	10 (0)	7.54 ± 0.11
16	3 (0)	2 (0)	10 (0)	7.45 ± 0.77
17	1 (−1)	0 (−1)	10 (0)	5.85 ± 0.91

Data are expressed as mean ± standard deviation (n = 3).

Table S2. Box–Behnken design scheme for moderate electric field fermentation and results of mucilage production by *Bacillus subtilis* HA.

Run	A: Frequency (log scale)	B: Current value (A)	C: Duty cycle (%)	Mucilage yield (%)
1	1.302	0.2	10	12.55 ± 0.46
2	2.802	0.2	50	13.33 ± 0.34
3	2.802	0.2	50	12.14 ± 0.39
4	4.301	0.1	50	8.74 ± 0.57
5	2.802	0.1	90	10.18 ± 0.51
6	4.301	0.2	90	10.23 ± 0.50
7	1.302	0.3	50	13.42 ± 0.59
8	4.301	0.3	50	9.10 ± 0.25
9	2.802	0.2	50	12.84 ± 0.43
10	1.302	0.2	90	12.37± 0.36
11	2.802	0.1	10	10.73± 1.07
12	4.301	0.2	10	8.80 ± 0.22
13	2.802	0.2	50	9.01 ± 0.21
14	2.802	0.2	50	13.72 ± 0.39
15	2.802	0.3	90	11.55 ± 0.62
16	1.302	0.1	50	12.14± 0.46
17	2.802	0.3	10	8.59 ± 0.34

Data are expressed as mean ± standard deviation (n = 3).

Table S3. Components of fermentation media to evaluate membrane-active reagents.

Components (mL)	DMSO	Tween 80	Glycerol
10% *Haematococcus pluvialis*		18.15	
40% glucose		11.93	
50% MSG[a)]		39.45	
Culture starter[b)]		7.50	
Sterilized water	72.82	72.52	69.97
DMSO[c)]	0.15	–	–
Tween 80	–	0.45	–
Glycerol	–	–	3.00
Total (w/v)		150	

[a)] MSG: monosodium L-glutamate

[b)] Culture starter: *Bacillus subtilis* HA cultured in 5% skim milk under shaking conditions for 24 h

[c)] DMSO: dimethyl sulfoxide
